# *Aucklandia lappa* Causes Cell Wall Damage in *Candida albicans* by Reducing Chitin and (1,3)-β-D-Glucan

**DOI:** 10.4014/jmb.2002.02025

**Published:** 2020-04-29

**Authors:** Heung-Shick Lee, Younhee Kim

**Affiliations:** 1 Department of Biotechnology and Bioinformatics, Korea University, Sejongsi 30019, Republic of Korea; 2 Department of Korean Medicine, Semyung University, Jecheon 27136, Republic of Korea

**Keywords:** *Aucklandia lappa*, Calcofluor white, *Candida albicans*, cell wall, chitin, (1, 3)-β-D-glucan

## Abstract

The fungal cell wall is a major target of antifungals. In this study, we report the antifungal activity of an ethanol extract from *Aucklandia lapp* against *Candida albicans*. We found that the extract caused cell wall injury by decreasing chitin synthesis or assembly and (1,3)-β-D-glucan synthesis. A sorbitol protection assay demonstrated that the minimum inhibitory concentration (MIC) of the *A. lappa* extract against *C. albicans* cells increased eight-fold from 0.78 to 6.24 mg/ml in 72 h. Cell aggregates, which indicate damage to the cell wall or membrane, were commonly observed in the *A. lappa*- treated *C. albicans* cells through microscopic analysis. In addition, the relative fluorescence intensities of the *C. albicans* cells incubated with the *A. lappa* extract for 3, 5, and 6 h were 92.1, 84.6, and 79.8%, respectively, compared to the controls, estimated by Calcofluor White binding assay. This result indicates that chitin content was reduced by the *A. lappa* treatment. Furthermore, synthesis of (1,3)-β-D-glucan polymers was inhibited to 84.3, 79.7, and 70.2% of that of the control treatment following incubation of *C. albicans* microsomes with the *A. lappa* extract at a final concentration equal to its MIC, 2× MIC, and 4× MIC, respectively. These findings suggest that the *A. lappa* ethanol extract may aid the development of a new antifungal to successfully control *Candida*- associated disease.

## Introduction

*Candida albicans* is an opportunistic, but principal, fungal pathogen of humans. Currently, infections by non- *albicans Candida* species, such as *C. glabrata*, *C. krusei,* C. *parapsilosis* and *C. tropicalis* are increasing in prevalence [1, 2]. The fungal cell wall surrounding the cell membrane provides cells with rigidity and protection against osmotic, dehydration, heat, and cold stress. It is also important for adhesion to host cells through the action of adhesins [3]. The fungal cell wall has coherent properties, due to its composition of microﬁbrillar polysaccharides and amorphous material made of other polysaccharides and proteins [4]. The inner layer of the cell wall of *C. albicans* is enriched with a chitin and polysaccharide matrix, while the outer layers are enriched with mannoprotein. Chitin is a linear polysaccharide made of *N*-acetylglucosamine connected by β-1,4-linkages [5]. Branched β-1,3-glucan is attached to β-1,6-glucan and chitin, and some chitin may be attached to β-1,6-glucan. Cell wall proteins are covalently linked to the meshwork of fibrillar polysaccharides [6].

Although intensive studies have been conducted to explore new antifungal drugs, the drugs currently available for the treatment of *Candida*-associated diseases are rather limited. This can be partly attributed to the similarities between human and fungal cells, due to their eukaryotic nature and close evolutionary relationship, as well as to the rise of resistant yeasts [7, 8]. Therefore, it is crucial to develop safe and effective antifungal drugs with low toxicity for humans. Plant products have been traditionally used in ethnomedicine, and many of these have been shown to be effective as antifungals or antimicrobials for the treatment of various diseases, as the plants from which they are derived contain various secondary metabolites with antimicrobial properties [9]. *Aucklandia lappa* Decne or *Saussurea lappa*, commonly known as costus or kuth, is a medicinal plant, and the dried roots of the plant have been traditionally used in Korea, China, and India for treating digestive disorders including nausea, vomiting, and diarrhea [10]. The plant extract is also reported to have anti-inflammatory, antibacterial, and anticancer activities [11]. The active compounds of *A. lappa* for anti-inflammatory activity include costunolide, dehydrocostus lactone, and alantolactone [12].

Here, we present an antifungal activity of the *A. lappa* ethanol extract against pathologically important *Candida* species including *C. albicans, C. glabrata, C. krusei,* and *C. tropicalis*. This study aimed to reveal the mode of antifungal action against *C. albicans,* which is a major model organism among fungal pathogens.

## Materials and Methods

### *Candida* Strains

The standard strain of *Candida albicans*, SC5314, was purchased from the American Type Culture Collection (USA). *C. glabrata* (ATCC 2001, KCCM 50044), *C. krusei* (ATCC 32196, KCCM 11426), and *C. tropicalis* (ATCC 750, KCCM 50075), obtained from the Korean Culture Center of Microorganisms (KCCM), were included as controls.

### Preparation of the *A. lappa* Ethanol Extract

Plant extracts from dried *A. lappa* roots were purchased from jchanbang.com, Korea. Thirty grams of the *A. lappa* roots were soaked in 300 ml of 70% ethanol for 1 h and centrifuged at 2,000 ×*g* for 20 min. The supernatant was concentrated using a vacuum evaporator and lyophilized to obtain an *A. lappa* ethanol extract. The ethanol extract was dissolved in dimethyl sulfoxide (DMSO) to 100 mg/ml, filter-sterilized, and kept at -20°C until used.

### Antifungal Susceptibility Test

The MICs of the *A. lappa* ethanol extract were determined by the standard broth microdilution CLSI M27-A3 protocol [13] with slight modification [14]. In all assays, DMSO was included as a growth and sterility control, and was the solvent for the *A. lappa* ethanol extract preparation. No inhibitory effect was detected with the solvent control up to a concentration of 1% (v/v). Amphotericin B (Sigma, USA) was included as a positive control.

### Neutral Red Staining

*C. albicans* SC5314 cells (5 × 10^6^ cells/ml) at the exponential phase were incubated with the *A. lappa* ethanol extract at a final concentration equivalent to its MIC in a YM medium (0.3% yeast extract, 0.3% malt extract, 1% peptone, and 2% dextrose) at 37°C, with shaking, for 2 h. The cells were harvested and stained with neutral red solution (Sigma) at a final concentration of 500 µg/ml for 5 min and examined by bright-field microscopy.

### Sorbitol Protection Assay

To investigate the effect of the *A. lappa* ethanol extract on the integrity of the *C. albicans* cell wall, a sorbitol protection assay was carried out using a modified CLSI M27-A3 protocol containing resazurin, as described above [15]. Briefly, two-fold serial dilutions of the *A. lappa* ethanol extract were prepared in two rows, and 0.8 M sorbitol was added to a row as an osmotic protectant. All the wells inoculated with *C. albicans* cell suspension were incubated at 35°C, and MICs were determined after 24 and 72 h.

### Spot Assay after Cell Wall Stress on *C. albicans*

*C. albicans* SC5314 cells (1 × 10^8^ cells/ml) were incubated with DMSO or the *A. lappa* extract in the absence or presence of 0.1% Congo Red (Sigma) or 0.025% Calcofluor White M2R (Sigma), with shaking, at 45°C. After 3 h, each culture was serially diluted ten-fold, and 4 µl of the undiluted and each serially diluted culture were spotted on YM plates. The plates were incubated at 37°C overnight, and photographed.

### Calcofluor White Staining and Fluorescence Microscopy

*C. albicans* SC5314 cells (5 × 10^6^ cells/ml) at the exponential phase were grown with DMSO or 0.78 mg/ml of the *A. lappa* ethanol extract, with shaking, at 37°C, and harvested after 5 and 6 h. The cells were washed with phosphate-buffered saline (PBS, pH 7.4) (Invitrogen, USA), and stained with 0.01% Calcofluor White in PBS for 5 min in the dark. The *C. albicans* cells were analyzed by fluorescence microscopy.

### Quantification of Chitin: Calcofluor White Binding Assay

Chitin was quantified using Calcofluor White binding assay. Exponential phase *C. albicans* SC5314 cells were grown in the absence or presence of the *A. lappa* ethanol extract at a final concentration equal to its MIC, with shaking, at 37°C. At regular intervals, 1 ml of each culture was harvested and washed with PBS (pH 7.4). The cell density of each group was then adjusted to 5 × 10^7^ cells/ml and cells were stained by resuspending the cell precipitate with 0.01% Calcofluor White in PBS. Each sample (100 µl) was aliquoted into a black, 96-well, flat- bottom microplate (BD Falcon, USA) in quadruplicate, and fluorescence intensities were immediately measured at 380 nm (20 nm width) excitation and 485 nm emission (20 nm width) wavelengths using a fluorometer (Tecan, Austria). The quantity of Calcofluor White binding to the *C. albicans* cell wall in the presence of the *A. lappa* extract was expressed as a percentage of the DMSO control.

### Preparation of Microsomes and (1,3)-β-D-Glucan Synthase Activity

Quantification of (1,3)-β-D-glucan synthase activity was performed by aniline blue assay with microsomal membranes. Microsomal membranes were prepared from exponential phase *C. albicans* SC5314 cells according to Shedletzky’s method [16] with slight modifications [15]. The protein concentration of the prepared microsomal membranes with apricot color was determined by Bradford microassay according to the manufacturer’s manual (Bio-Rad, USA), and stored at -27°C prior to use. The (1,3)-β-D-glucan synthase activity was measured based on the method of Frost *et al.* [17] with some modifications [15]. The (1,3)-β-D-glucan synthase activity assay was conducted with or without the *A. lappa* extract for 40 min at 25°C, and the synthesized glucans were stained specifically with aniline blue solution. Each reaction product was placed into a black, 96-well, flat-bottom microplate in quadruplicate, and fluorescence intensity was measured at 400 nm (20 nm width) excitation and 485 nm emission (20 nm width) wavelengths using a fluorometer. The quantity of (1,3)-β-glucan synthase activity in the presence of the *A. lappa* extract was represented as a percentage of the DMSO control. The data show the mean of quadruplicate measurements.

### Statistical Analysis

Each experiment was performed at least twice in quadruplicate, and the mean ± standard error for each treatment was calculated. The effect of *A. lappa* treatments when compared with controls was analyzed in SigmaPlot 13.0, using the Student’s *t*-test. A *p* value less than 0.05 was considered statistically significant.

## Results and Discussion

### Antifungal Susceptibility Test

People have used medicinal plants for the treatment of human diseases throughout human history. The advantages of plant extracts used in traditional medicine are their numerous and synergistic activities, caused by variable active ingredients, and confidence in their relatively high safety stemming from their centuries-long history of use [15]. In this study, we used the CLSI M27-A3 method with resazurin to demonstrate that the ethanol extract from *A. lappa* has an antifungal activity against *Candida* species. The overall range of MICs of the extract against the tested *Candida* species was between 98 and 780 µg/ml. The MIC of the *A. lappa* ethanol extract against *C. albicans* SC5314 was 780 µg/ml. The MIC of the *A. lappa* ethanol extract against *C. glabrata* ATCC 2001 was relatively low compared with the other tested *Candida* species, at 98 µg/ml. The control antifungal, amphotericin B, inhibited the growth of the tested *Candida* species at a concentration of 1 µg/ml (Table 1).

### Neutral Red Staining

**Fig. 1:**
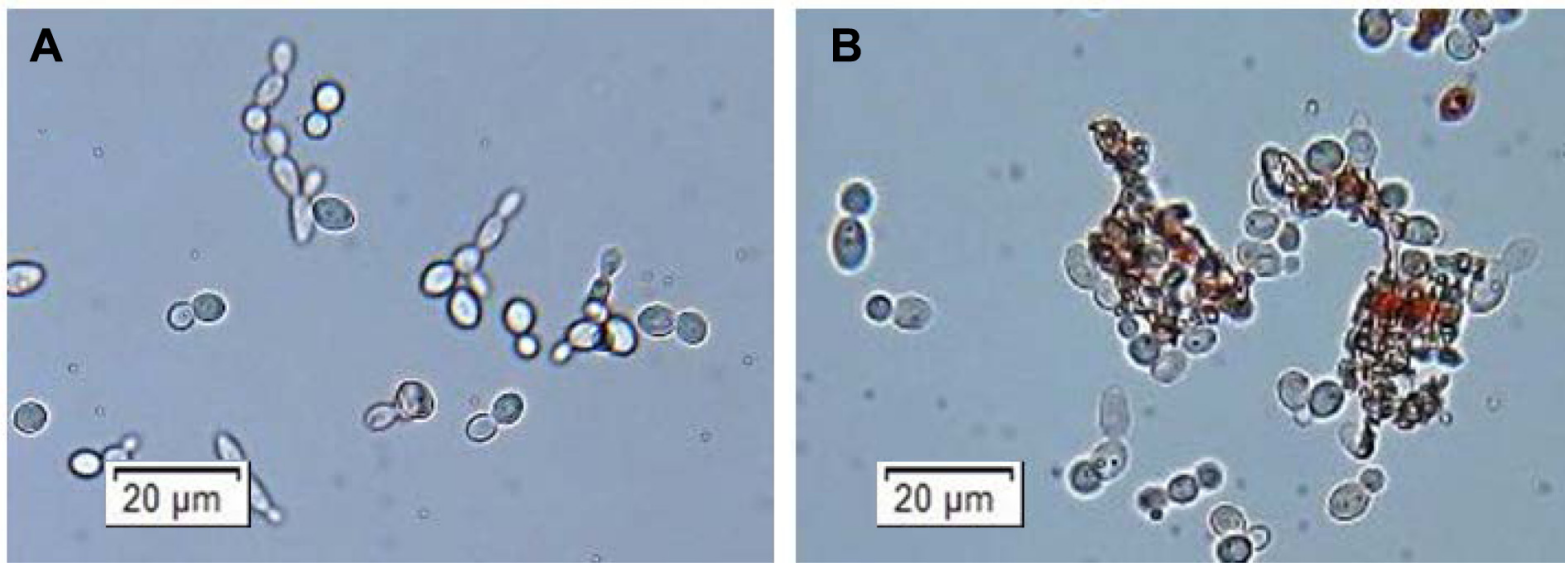
Neutral red-stained *C. albicans* cells. *C. albicans* SC5314 cells at the exponential phase were incubated with DMSO (A) or the ethanol extract of *A. lappa* at a concentration of 0.78 mg/ml (B), with shaking, at 37°C for 2 h. The cells were harvested, stained with neutral red (500 mg/µl) and examined by bright-field microscopy. Stained cells are dead and therefore have damaged vacuolar membranes.

*C. albicans* SC5314 cells at the log phase were incubated with the *A. lappa* extract at its MIC for 2 h, and the cells stained with neutral red were examined using a bright-field microscope. While neutral red readily penetrates the yeast cell membrane and enters vacuoles with acidic pH to stain red in live cells, it leaks out into the cytoplasm in dead cells, where vacuolar membranes are damaged. Therefore, cytoplasm is stained with neutral red in dead yeast cells, but in live cells only the vacuoles are stained [18]. The *C. albicans* cells looked normal, being ovoid, and were not stained with neutral red in the DMSO-treated control (Fig. 1A), but some of the *A. lappa*-treated cells were stained dark red in their cytoplasm (Fig. 1B). In addition, *C. albicans* cells that had been incubated with the *A. lappa* extract for 2 h displayed a series of characteristic alterations, such as disruption and formation of cell aggregates, which are indicative of cell membrane or cell wall damage.

### Sorbitol Protection Assay

Damage of the essential cell wall components by antifungals will cause cell lysis, but cells can stay alive with an appropriate osmotic stabilizer in the medium [17]. To investigate whether the antifungal activity of *A. lappa* is linked to the modification of the fungal cell wall, CLSI M27-A3 microdilution assay was carried out with the *A. lappa* extract against *C. albicans* cells with or without 0.8 M sorbitol (Table 2). Regardless of the sorbitol, the MIC values of *A. lappa* against *C. albicans* cells remained constant at 0.78 mg/ml after 24 h. However, the MIC value of the *A. lappa* extract against *C. albicans* cells increased eight-fold to 6.24 mg/ml in RPMI1640 medium supplemented with sorbitol after 72 h of incubation, compared to that in the medium without sorbitol. Therefore, the increased MIC value in the sorbitol protection assay indicates that the *A. lappa* ethanol extract is involved in disturbing cell wall integrity in *C. albicans*. The results from neutral red staining and sorbitol protection assay suggest that the *A. lappa* ethanol extract causes changes in the composition and structure of the *C. albicans* cell wall.

### Spot Assay after Cell Wall Stress on *C. albicans*

**Fig. 2:**
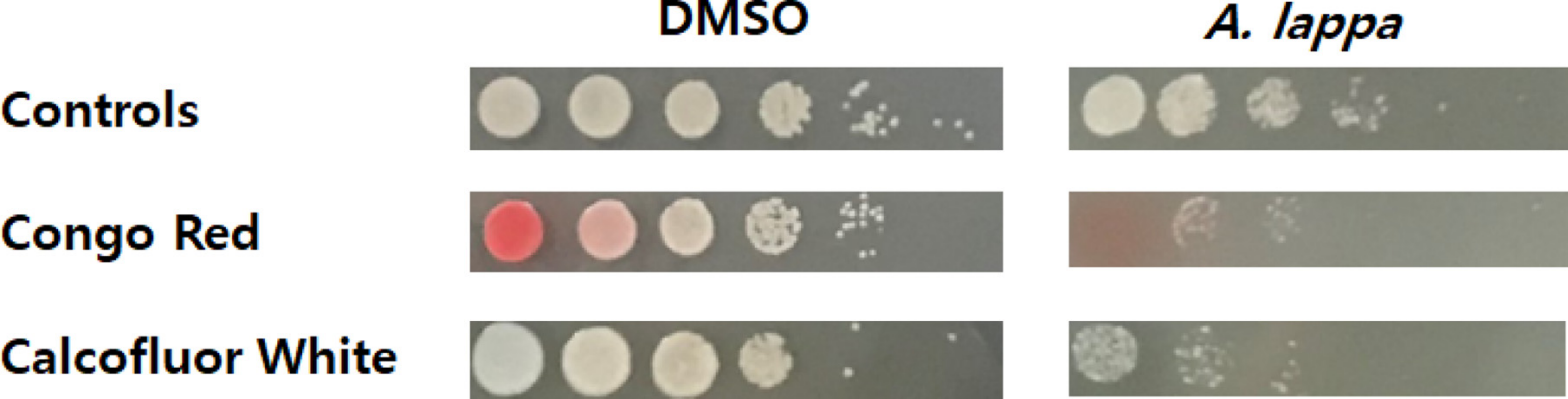
Spot assay after cell wall stress on *C. albicans*. *C. albicans* SC5314 cells (1 × 10^8^ cells/ml) were grown in YM broth with DMSO or the *A. lappa* ethanol extract at a final concentration of its minimum inhibitory concentration at 45°C for 3 h in the absence or presence of 0.1% Congo Red and 0.025% Calcofluor White, respectively. Each culture was serially diluted ten- fold, and 4 µl of the undiluted and diluted cultures were sequentially spotted on YM plates and grown overnight at 37°C. The plates were photographed.

In terms of biomass, (1,3)-β-glucan and mannoproteins are the major components of the cell wall, followed by (1,6)-β-glucan and chitin [19]. Congo red and Calcofluor White are known to be cell wall disturbing agents: They interact with various polysaccharides, but show a high affinity for chitin and cellulose, predominantly. In particular, both dyes inhibited chitin and (1,3)-β-D-glucan synthases in isolated *Geotrichum lactis* cell-free systems, and changed the assembly of chitin microfibrils in yeasts [20]. On the other hand, the human fungal pathogen *C. albicans* can withstand and grow under conditions many other fungi cannot survive, such as high temperatures of up to 45°C [21]. The elevated temperature increases the membrane fluidity and influences the membrane composition, which in turn affect the polysaccharide composition of the cell wall. In fact, it is reported that cells respond to cell wall stress by increasing the chitin levels in the cell wall to strengthen it [22]. Since the *A. lappa* ethanol extract damages cell wall integrity similar to high temperature or cell wall-perturbing agents, we investigated whether the *A. lappa* extract has any fatal or lethal effects on *C. albicans* cells when exposed to Congo red or Calcofluor White at a high temperature (45°C) for 4 h. As seen in Fig. 2, *C. albicans* SC5314 cells grew well at 45°C and the antifungal effect of *A. lappa* was fairly good at that temperature, because the number of *C. albicans* colonies was less than 10% of that of the DMSO control. Congo red (0.1%) hindered the growth of the DMSO control, but the effect was small. However, the effect of Congo red was serious when accompanied by the *A. lappa* extract. The antifungal effect of 0.025% Calcofluor White was fair on the DMSO control, but the effect on *C. albicans* was greatly enhanced when the *A. lappa* extract was also present. The results displayed in Fig. 2 show the possibility that the *A. lappa* ethanol extract is involved in weakening the *C. albicans* cell wall by altering chitin and (1,3)-β-D-glucan formation.

### Calcofluor White Staining and Fluorescence Microscopy

**Fig. 3:**
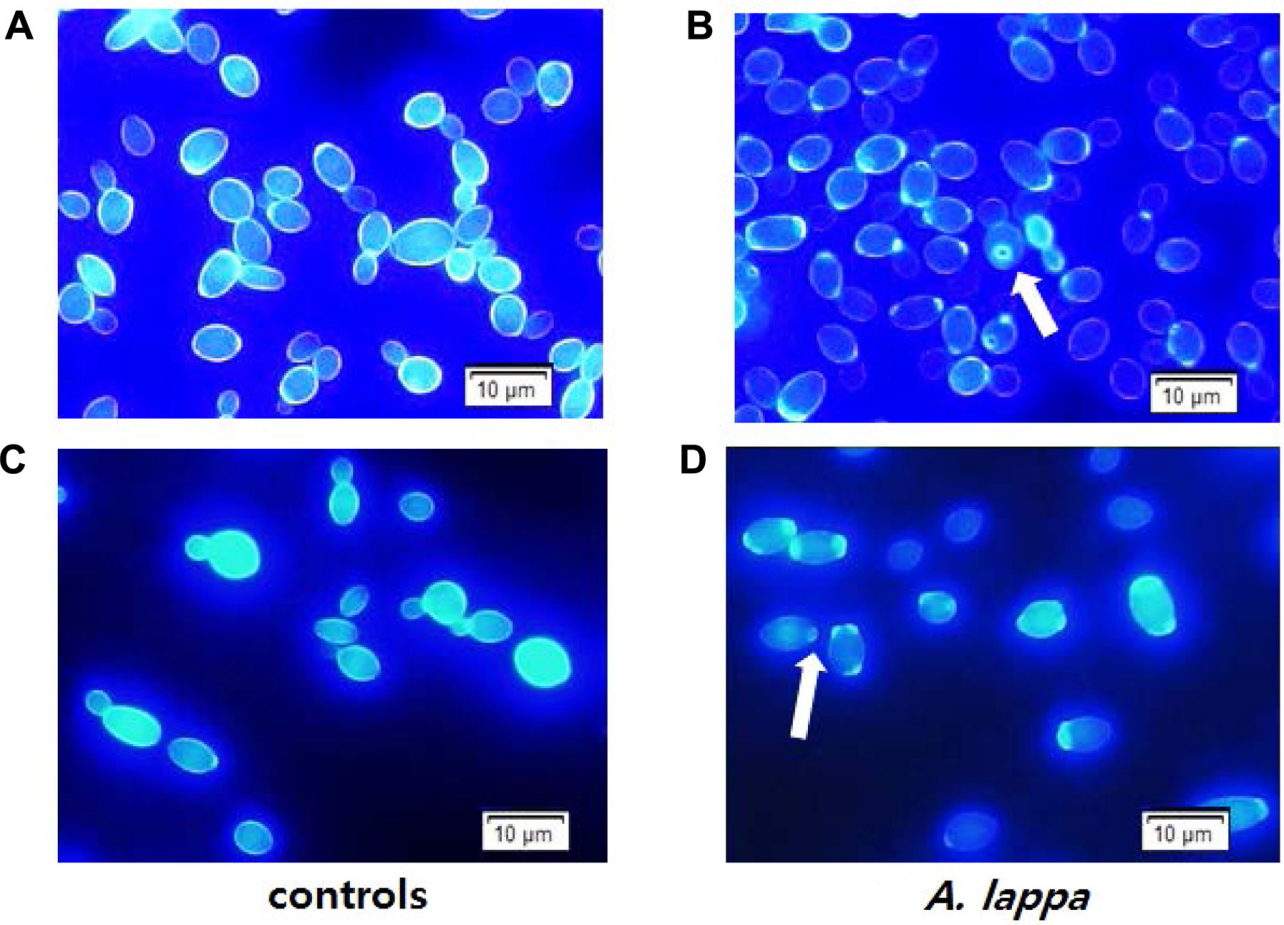
Calcofluor White staining. The exponential phase *C. albicans* SC5314 cells were treated with DMSO or the *A. lappa* ethanol extract at a concentration of its minimum inhibitory concentration, with shaking, at 37°C for 5 h (**A** and **B**, respectively) and 6 h (**C** and **D**, respectively). The cells were washed with PBS (pH 7.4), stained with 0.01% Calcofluor White in PBS for 5 min in the dark, and examined by a fluorescence microscope equipped with WU fluorescence filter cube. Arrows indicate bud scars.

Calcofluor White is a dye that ﬂuoresces with an intense blue color when excited under ultraviolet light. It predominantly stains the chitin layer in the cell walls of fungi [23], and bud scars intensively in budding yeasts, which contain a high concentration of chitin. *C. albicans* cells grown with DMSO or the *A. lappa* ethanol extract for either 5 or 6 h were stained with Calcofluor White, and observed by fluorescence microscopy. There were some differences between the DMSO controls and the *A. lappa*-treated *C. albicans* cells (Fig. 3): first, (Note: 'firstly, secondly, thirdly' not commonly used in modern NA English) bud scars, crater-like rings of scar tissue formed in the mother cell following cytokinesis [24], were particularly noticeable in the *A. lappa*-treated *C. albicans* compared to the DMSO controls (Figs. 3B and 3D). Second, the cell walls of the *A. lappa*-treated *C. albicans* appeared thinner than those of the DMSO controls. Third, photobleaching, fading of the blue fluorescence of Calcofluor White, was more obvious in the *A. lappa*-treated *C. albicans* cells than in the controls. Hence, the thinner cell walls and remarkable photobleaching observed in the *A. lappa*-treated *C. albicans* cells are thought to be linked to the low amount of chitin in the cells. Fourth, cells were variable in size due to the ongoing budding process in the DMSO controls, while cells were rather similar in size, probably due to inhibition of the budding process, in the *A. lappa*-treated *C. albicans* cells. Based on the data, it is hypothesized that the *A. lappa*-treated *C. albicans* cells have a thinner cell wall with loose chitin, and the treatment causes a series of events to take place such as the inhibition of the budding process and cell cycle arrest.

### Quantification of Chitin Content with Calcofluor White Binding

**Fig. 4:**
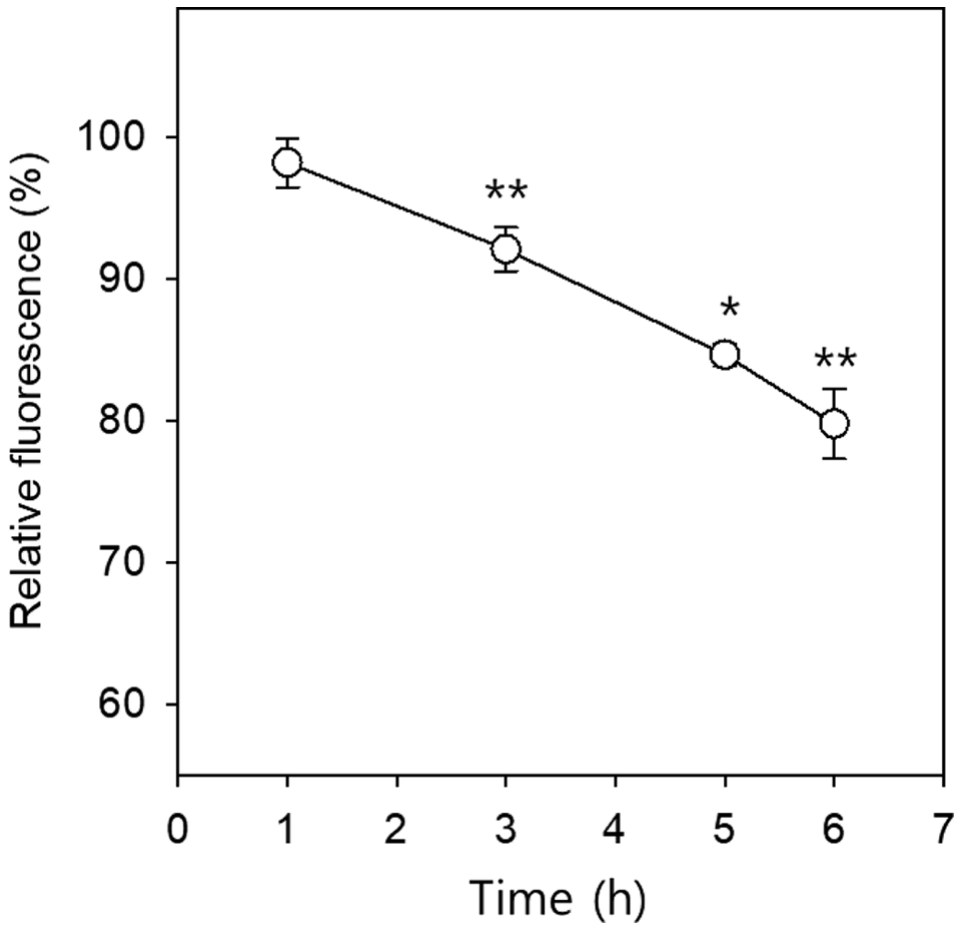
Calcofluor White binding assay. *C. albicans* SC5314 cells (5 × 10^7^ cells/ml) were incubated with DMSO or 0.78 mg/ml of the *A. lappa* ethanol extract, with shaking, at 37°C and 1 ml of each culture was harvested at the indicated time. The cells were washed with PBS (pH 7.4) and the cell density of each group was adjusted to 5 × 10^7^ cells/ml and stained with 0.01% Calcofluor White in PBS. Aliquots of 100 μl of each sample were placed into a black, 96-well, flat-bottom microplate in quadruplicate, and the fluorescence intensity was measured. The quantity of Calcofluor White binding to the *C. albicans* cell wall in the presence of the *A. lappa* extract was expressed as a percentage of the DMSO control. The data represent the mean of the quadruplicate measurements ± SE. * *p* < 0.05, **p < 0.01.

In terms of dry mass, (1,3)-β-glucan and (1,6)-β-glucan make up 40% and 20% of the cell wall [19]. Chitin, a linear polymer of N-acetylglucosamine, makes up 2% of *C. albicans* cell wall biomass [5]. Calcofluor White binds to the (1,3)-β-glucan and chitin of the fungal cell wall, and the intensity of Calcofluor White fluorescence in yeast cells was found to be an accurate reflection of the relative chitin content [25]. To compare the chitin content of *A. lappa*-treated *C. albicans* cells to that of controls, fluorescence intensity was measured by fluorometry after Calcofluor White binding assay was performed. The relative fluorescence intensities of the *C. albicans* cells incubated with the *A. lappa* extract for 1, 3, 5, and 6 h were 97.8, 92.1, 84.6, and 79.8%, respectively, compared to the controls, as estimated by Calcofluor White binding assay (Fig. 4). The relative fluorescence intensity of the* A. lappa*-treated cells decreased in a time-dependent manner, and the differences in fluorescence intensity between the DMSO control and each experimental group were statistically significant between 3 and 6 h (*p* < 0.05). The data suggest that the *C. albicans* cell wall becomes thinner through a reduction in chitin synthesis or assembly, caused by *A. lappa* treatment. Based on the data from the sorbitol protection assay and Calcofluor White binding assay, it can be concluded that chitin synthesis or assembly in the cell wall is a target of the *A. lappa* ethanol extract in *C. albicans*.

The classical method of measuring cell wall chitin content based on glucosamine release through extensive acid hydrolysis is painstaking and time-consuming. To quantify chitin levels in fungi, both epifluorescence microscopy and flow cytometric methods are feasible following staining with Calcofluor White [26]. The Calcofluor White binding assay that we have developed in this paper is an alternative, simple, rapid, and reliable method of measuring chitin content in cell walls.

### Effect of *A. lappa* on (1,3)-β-D-Glucan Synthase

**Fig. 5:**
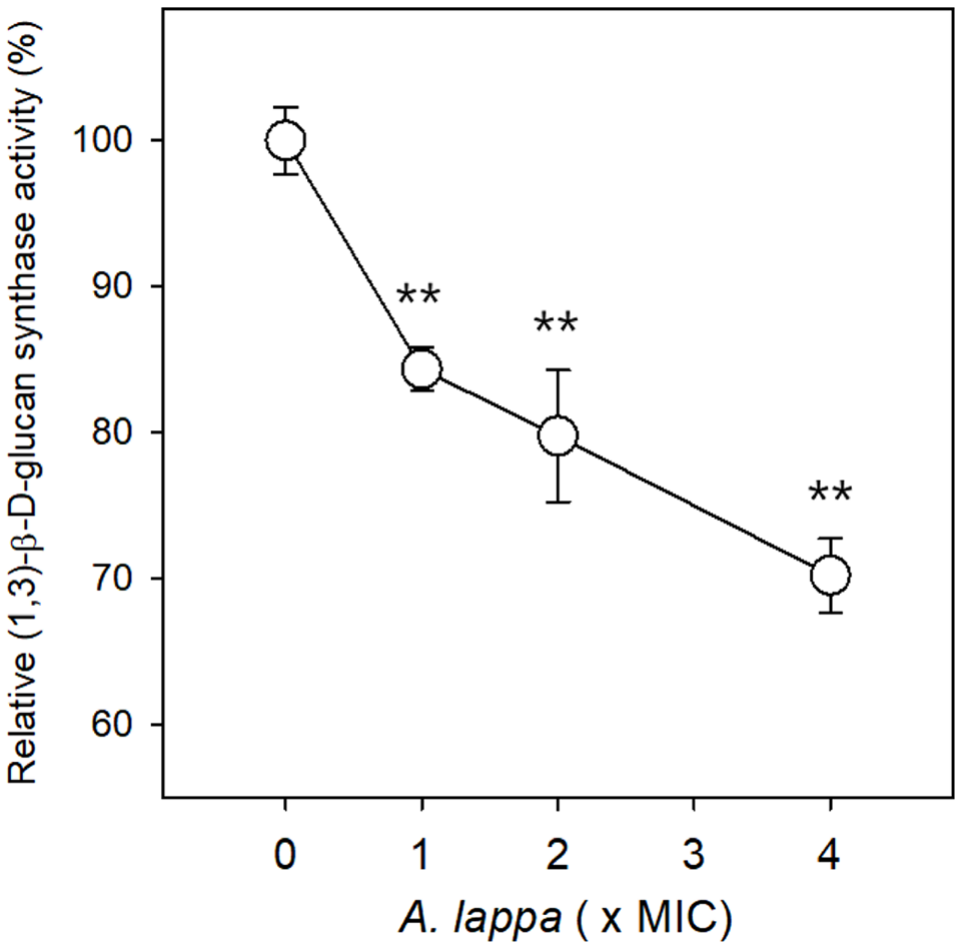
Effect of the *Aucklandia lappa* extract on (1,3)-β-D-glucan synthase activity. Quantification of (1,3)-β-D- glucan synthase activity was performed using aniline blue assay with *C. albicans* SC5314 microsomal membranes. The (1,3)-β- D-glucan synthase activity assay was performed with or without the *A. lappa* extract for 40 min at 25°C, and the synthesized glucans were stained with aniline blue solution. The reaction products were placed into a black, 96-well, flat-bottom microplate in quadruplicate, and the fluorescence intensity was measured. The quantity of (1,3)-β-glucan synthase activity in the presence of the *A. lappa* extract was expressed as a percentage of the DMSO control. The data represent the mean of quadruplicate measurements ± SE. **p < 0.01.

(1,3)-β-D-glucan is the primary polysaccharide in the fungal cell wall. It is synthesized by glucan synthase located in the cell membrane. (1,3)-β-D-glucan synthase is considered a molecular target in the search for compounds with potential antifungal activity. Therefore, it was examined whether the *A. lappa* ethanol extract also inhibits the (1,3)-β-D-glucan synthase of *C. albicans* cells. (1,3)-β-D-glucan synthase activity was measured with microsomal membranes prepared from *C. albicans* SC5314 cells by aniline blue assay. Yeast microsomes are vesicular structures with variable sizes that originate from the endoplasmic reticulum or fragmented cell membranes, and contain soluble enzymes such as (1,3)-β-D-glucan synthases [17]. Microsomal membranes and substrates for (1,3)-β-D-glucan synthase were incubated with or without the *A. lappa* extract at 25°C for 40 min, and the newly formed (1,3)-β-D-glucan was measured using aniline blue assay. Fluorescence intensity representing (1,3)-β-D-glucan synthase activity in the presence of the *A. lappa* extract was expressed as percentages, and the value in the absence of the *A. lappa* extract was regarded as 100% (Fig. 5). Synthesis of (1,3)-β- D-glucan was inhibited to 84.3, 79.7, and 70.2% of that of the control treatment following treatment of microsomes with the *A. lappa* extract at a final concentration equal to its MIC, 2× MIC, and 4× MIC, respectively, and the differences between the DMSO control and the *A. lappa*-treated *C. albicans* cells were statistically significant (*p* < 0.01).

As mentioned previously, (1,3)-β-glucan and chitin occupy 40% and 2% of total cell wall biomass. Our data demonstrate the *A. lappa* ethanol extract decreases chitin content and inhibits (1,3)-β-D-glucan synthase activity. Therefore, it can be concluded that the *A. lappa* extract considerably damages the *C. albicans* cell wall and has therapeutic potential against *Candida*-associated infections.

## Figures and Tables

**Table 1:** Minimum inhibitory concentrations (MICs) of the *Aucklandia lappa* ethanol extract against *Candida* spp.

	MIC (µg/ml)	

	*A. lappa*	Amphotericin B
*C. albicans* SC5314	780	1
*C. krusei* ATCC 32196	390	1
*C. glabrata* ATCC 2001	98	1
*C. tropicalis* ATCC 750	780	1

Antifungal susceptibilities of *Candida* species were determined by the modified CLSI M27-A3 method containing resazurin.

**Table 2:** Sorbitol protection assay.

Incubation time (h)	MIC (mg/ml)	

	No sorbitol	Sorbitol
24	0.78	0.78
72	0.78	6.24

Antifungal susceptibility tests were carried out according to the modified CLSI M27-A3 protocol containing resazurin with or without 0.8 M sorbitol as an osmotic stabilizer. The 96-well round-bottom plate was incubated at 35°C, and minimum inhibitory concentrations (MICs) were determined after 24 and 72 h, respectively.

## References

[1] Krcmery V, Barnes AJ (2002). Non-albicans *Candida* spp. causing fungaemia: pathogenicity and antifungal resistance. J. Hosp. Infect..

[2] Hachem R, Hanna H, Kontoyiannis D, Jiang Y, Raad I (2008). The changing epidemiology of invasive candidiasis: *Candida glabrata* and Candida krusei as the leading causes of candidemia in hematologic malignancy. Cancer.

[3] Reyna-Beltrán E, Méndez CIB, Iranzo M, Mormeneo S, Luna-Arias JP (2019). The cell wall of *Candida albicans*: A proteomics view. IntechOpen Chapter.

[4] Ruiz-Herrera J, Ortiz-Castellanos L (2010). Analysis of the phylogenetic relationships and evolution of the cell walls from yeasts and fungi. FEMS Yeast Res..

[5] Ruiz-Herrera J, Elorza MV, Valentín E, Sentandreu R (2006). Molecular organization of the cell wall of *Candida albicans* and its relation to pathogenicity. FEMS Yeast Res..

[6] Chaffin WL (2008). *Candida albicans* cell wall proteins. Microbiol. Mol. Biol. Rev..

[7] Carson CF, Mee BJ, Riley TV (2002). Mechanism of action of *Melaleuca alternifolia* (tea tree) oil on Staphylococcus aureus determined by time-kill, lysis, leakage, and salt tolerance assays and electron microscopy. Antimicrob. Agents Chemother..

[8] Shapiro RS, Robbins N, Cowen LE (2011). Regulatory circuitry governing fungal development, drug resistance, and disease. Microbiol. Mol. Biol. Rev..

[9] Perumal S, Ramar, Gopalakrishnakone P (2010). Therapeutic potential of plants as anti-microbials for drug discovery. Evid. Based Complement. Alternat. Med..

[10] Li A, Sun A, Liu R (2005). Preparative isolation and purification of costunolide and dehydrocostuslactone from *Aucklandia lappa* Decne by high-speed counter-current chromatography. J. Chromatogr. A.

[11] Kamalpreet LK, Singh A, Kaur J, Kaur N (2019). A brief review of remedial uses of *Saussurea lappa*. J. Pharmacogn. Phytochem..

[12] Seo CS, Lim HS, Jeong SJ, Shin HK (2015). Anti-allergic effects of sesquiterpene lactones from the root of *Aucklandia lappa* Decne. Mol. Med. Rep..

[13] Clinical and Laboratory Standards Institute (2008). M27-A3. Reference method for broth dilution antifungal susceptibility testing of yeasts: Approved standard.

[14] Liu M, Seidel V, Katerere DR, Gray AI (2007). Colorimetric broth microdilution method for the antifungal screening of plant extracts against yeast. Methods.

[15] Lee HS, Kim Y (2016). Antifungal activity of Salvia miltiorrhiza against *Candida albicans* is associated with the alteration of membrane permeability and (1,3)-β-D-glucan synthase activity. J. Microbiol. Biotechnol..

[16] Shedletzky E, Unger C, Delmer DP (1997). A microtiter-based fluorescence assay for (1,3)-β-glucan synthases. Anal. Biochem..

[17] Frost DJ, Brandt KD, Cugier D, Goldman R (1995). A whole-cell *Candida albicans* assay for the detection of inhibitors towards fungal cell wall synthesis and assembly. J. Antibiot..

[18] Kolotila MP, Smith CW, Rogers AL (1987). Candidacidal activity of macrophages from three mouse strains as demonstrated by a new method: neutral red staining. J. Med. Vet. Mycol..

[19] Lesage G, Bussey H (2006). Cell wall assembly in *Saccharomyces cerevisiae*. Microbiol. Mol. Biol. Rev..

[20] Roncero C, Duran A (1985). Effect of Calcofluor white and Congo red on fungal cell wall morphogenesis: *in vivo* activation of chitin polymerization. J. Bacteriol..

[21] Heilmann CJ, Sorgo AG, Mohammadi S, Sosinska GJ, de Koster CG, Brul S (2013). Surface stress induces a conserved cell wall stress response in the pathogenic fungus *Candida albicans*. Eukaryot. Cell.

[22] Lee KK, Maccallum DM, Jacobsen MD, Walker LA, Odds FC, Gow NA (2012). Elevated cell wall chitin in *Candida albicans* confers echinocandin resistance *in vivo*. Antimicrob. Agents Chemother..

[23] Brasch J, Kreiselmaier I, Christophers E (2003). Inhibition of dermatophytes by optical brighteners. Mycoses.

[24] Barton AA (1950). Some aspects of cell division in *Saccharomyces cerevisiae*. J. Gen. Microbiol..

[25] Walker LA, Munro CA, De Bruijn I, Lenardon MD, McKinnon A, Gow NA (2008). Stimulation of chitin synthesis rescues *Candida albicans* from echinocandins. PLoS pathog..

[26] Costa-de-Oliveira S, Silva AP, Miranda IM, Salvador A, Azevedo MM, Munro CA (2013). Determination of chitin content in fungal cell wall: an alternative flow cytometric method. Cotometry.

